# Risk of COVID-19 hospital admission among children aged 5–17 years with asthma in Scotland: a national incident cohort study

**DOI:** 10.1016/S2213-2600(21)00491-4

**Published:** 2022-02

**Authors:** Ting Shi, Jiafeng Pan, Srinivasa Vittal Katikireddi, Colin McCowan, Steven Kerr, Utkarsh Agrawal, Syed Ahmar Shah, Colin R Simpson, Lewis Duthie Ritchie, Chris Robertson, Aziz Sheikh, Public Health Scotland and the EAVE II Collaborators

**Affiliations:** a Usher Institute, Edinburgh Medical School, University of Edinburgh, Edinburgh, UK; b Department of Mathematics and Statistics, University of Strathclyde, Glasgow, UK; c MRC/CSO Social and Public Health Sciences Unit, University of Glasgow, Glasgow, UK; d School of Medicine, University of St Andrews, St Andrews, UK; e School of Health, Wellington Faculty of Health, Victoria University of Wellington, Wellington, New Zealand; f School of Medicine and Dentistry, University of Aberdeen, Aberdeen, UK; g Public Health Scotland, Glasgow, UK; h Asthma UK Centre for Applied Research, Usher Institute, University of Edinburgh, Edinburgh, UK

## Abstract

**Background:**

There is an urgent need to inform policy deliberations about whether children with asthma should be vaccinated against SARS-CoV-2 and, if so, which subset of children with asthma should be prioritised. We were asked by the UK's Joint Commission on Vaccination and Immunisation to undertake an urgent analysis to identify which children with asthma were at increased risk of serious COVID-19 outcomes.

**Methods:**

This national incident cohort study was done in all children in Scotland aged 5–17 years who were included in the linked dataset of Early Pandemic Evaluation and Enhanced Surveillance of COVID-19 (EAVE II). We used data from EAVE II to investigate the risk of COVID-19 hospitalisation among children with markers of uncontrolled asthma defined by either previous asthma hospital admission or oral corticosteroid prescription in the previous 2 years. A Cox proportional hazard model was used to derive hazard ratios (HRs) and 95% CIs for the association between asthma and COVID-19 hospital admission, stratified by markers of asthma control (previous asthma hospital admission and number of previous prescriptions for oral corticosteroids within 2 years of the study start date). Analyses were adjusted for age, sex, socioeconomic status, comorbidity, and previous hospital admission.

**Findings:**

Between March 1, 2020, and July 27, 2021, 752 867 children were included in the EAVE II dataset, 63 463 (8·4%) of whom had clinician-diagnosed-and-recorded asthma. Of these, 4339 (6·8%) had RT-PCR confirmed SARS-CoV-2 infection. In those with confirmed infection, 67 (1·5%) were admitted to hospital with COVID-19. Among the 689 404 children without asthma, 40 231 (5·8%) had confirmed SARS-CoV-2 infections, of whom 382 (0·9%) were admitted to hospital with COVID-19. The rate of COVID-19 hospital admission was higher in children with poorly controlled asthma than in those with well controlled asthma or without asthma. When using previous hospital admission for asthma as the marker of uncontrolled asthma, the adjusted HR was 6·40 (95% CI 3·27–12·53) for those with poorly controlled asthma and 1·36 (1·02–1·80) for those with well controlled asthma, compared with those with no asthma. When using oral corticosteroid prescriptions as the marker of uncontrolled asthma, the adjusted HR was 3·38 (1·84–6·21) for those with three or more prescribed courses of corticosteroids, 3·53 (1·87–6·67) for those with two prescribed courses of corticosteroids, 1·52 (0·90–2·57) for those with one prescribed course of corticosteroids, and 1·34 (0·98–1·82) for those with no prescribed course, compared with those with no asthma.

**Interpretation:**

School-aged children with asthma with previous recent hospital admission or two or more courses of oral corticosteroids are at markedly increased risk of COVID-19 hospital admission and should be considered a priority for vaccinations. This would translate into 9124 children across Scotland and an estimated 109 448 children across the UK.

**Funding:**

UK Research and Innovation (Medical Research Council), Research and Innovation Industrial Strategy Challenge Fund, Health Data Research UK, and Scottish Government.

## Introduction

Of all the COVID-19 cases in the UK, approximately 9% have been in school-aged children (aged 5–17 years).^1^ Although most COVID-19 cases in children are mild, some children might need to be admitted to hospital or the illness can lead to death.^2^ Approximately 18% of children admitted to hospital needed critical care.^2^ In the face of considerable uncertainty about the benefits and risks of COVID-19 vaccines in children, and concerns about limited vaccine supplies in many parts of the world, policy responses to vaccinating children have differed quite markedly. Thus, identifying which group of school-aged children might benefit from earlier doses of the COVID-19 vaccines has important implications for vaccine delivery worldwide.^3^, ^4^ Identifying this group could help to reduce the risk of infection and consequently the need for children to have time off school; and might also reduce the risk of spread of SARS-CoV-2 within schools and households. The UK's Joint Commission on Vaccination and Immunisation (JCVI) has not recommended universal vaccination of children and young people. Rather, the JCVI's policy was first to recommend COVID-19 vaccination to all children aged 16–17 years and then to offer vaccines to children aged 12–15 years with severe neurodisabilities, Down syndrome, immunosuppression, and multiple or severe learning disabilities.^5^

**Research in context**

**Evidence before this study**

Understanding which children with asthma are at increased risk of serious COVID-19 outcomes is of critical importance in deliberations on vaccine prioritisation. We searched PubMed, medRxiv, and SSRN for observational studies, with no language restrictions, using the terms “SARS-CoV-2”, “COVID-19”, “children”, “adolescents”, “young adults”, and “asthma”, for studies published between March 1, 2020, and Oct 7, 2021. We did not identify any studies investigating the risk of severe COVID-19 outcomes among children with varying severity or control of asthma. Previous work has shown that adults with poorly controlled asthma are at increased risk of serious COVID-19 outcomes.

**Added value of this study**

To our knowledge, this is the first national study assessing the risk of COVID-19 hospital admissions in children aged 5–17 years with markers of uncontrolled asthma. We found that children with poorly controlled asthma (defined by either previous asthma hospital admission or oral corticosteroids prescription) had an increased risk of COVID-19 hospital admission compared with those with well controlled asthma or no asthma. The hazard ratio was significant after adjusting for age, sex, socioeconomic status, comorbidity, and previous hospital admission.

**Implications of all the available evidence**

We provide national evidence that children and young people aged 5–17 years with poorly controlled asthma are at increased risk of COVID-19 hospital admission. These findings have been used to inform deliberations on which children aged 12–15 years to prioritise for COVID-19 vaccination. Consideration needs to be given to extending the offer of vaccination to younger children.

An estimated 5·4 million people in the UK are receiving treatment for asthma, including 1·1 million children.^6^ A cross-sectional study of more than 43 000 children in the USA found that among children with a COVID-19 diagnosis, asthma was the most common diagnosed condition.^7^ There is considerable uncertainty about whether children with asthma should be vaccinated against SARS-CoV-2 and, if so, who should be prioritised for vaccination. There is a scarcity of research on COVID-19 and asthma in children despite evidence of the clinical significance of asthma in adults with COVID-19.^8^ A Brazilian observational study of 607 children with clinically diagnosed COVID-19 indicated that asthma was associated with lower respiratory tract involvement and worse COVID-19 severity scores among those seeking emergency room care.^9^ Evidence for children with asthma being at the highest risk of severe COVID-19 outcomes is limited as there have been no population-based analyses.

There has been uncertainty about vaccinating children with asthma in the UK, partially due to the low absolute risks of acute severe sequelae from COVID-19 in children, the risk of vaccine side-effects, and concerns about vaccine supplies, particularly in the context of ongoing deliberations on vaccine booster doses for adults. Given that asthma is one of the most prevalent long-term conditions in childhood, in response to a request from JCVI, we sought to investigate the risk of hospital admission from COVID-19 among school-aged children with markers of uncontrolled asthma.

## Methods

### Study design and participants

This national incident cohort study was done in all children in Scotland aged 5–17 years who were included in the linked dataset of Early Pandemic Evaluation and Enhanced Surveillance of COVID-19 (EAVE II). EAVE II is a Scotland-wide COVID-19 surveillance platform that has been used to track and forecast the epidemiology of COVID-19, inform risk stratification assessment, and investigate vaccine effectiveness and safety.^10^, ^11^, ^12^, ^13^, ^14^ It comprises national health-care datasets for 5·4 million people (approximately 99% of the Scottish population) deterministically linked through the Community Health Index (CHI) number, which is a unique identifier used in all health-care contacts across National Health Service (NHS) Scotland. We used data from EAVE II to describe the demographic profile of children with asthma who had SARS-CoV-2 infections and COVID-19 hospital admissions. We also undertook a national incident cohort analysis to investigate risks of hospital admission in children with asthma, stratified by markers of asthma control (previous admission to hospital due to asthma or previous oral corticosteroid prescription).

The cohort was set up on March 1, 2020 (retrospectively assigned as it was shortly before the first person was admitted to hospital due to COVID-19 in Scotland).^15^ All individuals were followed up from March 1, 2020, until the date of COVID-19 related hospitalisation, date of death, or end of follow-up (July 27, 2021), whichever came first.

Ethical approval was obtained from the National Research Ethics Service Committee, Southeast Scotland 02 (reference number, 12/SS/0201). The Public Benefit and Privacy Panel Committee of Public Health Scotland approved the linkage and analysis of the de-identified datasets for this project (1920-0279).

### Data sources and procedures

The national datasets linked using CHI number were the Electronic Communication of Surveillance in Scotland (national database for all virology testing), primary care (demographics and clinical history), the Scottish Morbidity Record (which records hospitalisations), National Records of Scotland (which records mortality data), and Prescribing Information System (for prescription data). A data linkage diagram is shown in the appendix (p 15).

Asthma and other variables of interest were measured on March 1, 2020, and defined by the QCOVID risk prediction algorithm, which consists of 30 clinical characteristics identified from primary care records that are known to be associated with increased risk of serious COVID-19 outcomes in adults (appendix p 1).^16^ We excluded risk groups that were either not relevant to the paediatric population (ie, resident in a care home or homeless [no children were classified as homeless over the study period], chronic obstructive pulmonary disease, coronary heart disease, dementia, and Parkinson's disease) or that had substantial missing data (body-mass index and ethnicity; proportion of missing data are shown in appendix p 3). We only included the predefined risk group if there were at least five COVID-19 hospital admissions within a risk group during the study period. This resulted in nine risk groups in addition to asthma being included and analysed as potential confounders (appendix p 3). The comparison group of children without asthma might have other chronic comorbidities.

We also assessed the risk of COVID-19 hospital admissions stratified by two markers of asthma control. First, we used hospital admission for asthma before March 1, 2020. This included all hospitalisations with primary admission diagnosis based on International Classification of Diseases Tenth Revision (ICD-10) codes J45 and J46 within 2-years before March 1, 2020. Second, we used previous oral corticosteroids prescription (prednisolone) as the marker of asthma control. The current QCOVID prediction algorithm definition for poorly controlled asthma is having three or more prescribed courses of corticosteroids in the preceding 12 months. To capture prescriptions comprehensively, we looked back 2 years before March 1, 2020, for prednisolone prescriptions in children and compared the risk of COVID-19 hospital admission for those with one, two, three or more, or no courses of corticosteroids within the 24-month period.

Building on methods that have previously been described in detail,^13^, ^17^ we defined individuals who tested positive with real-time RT-PCR as having SARS-CoV-2 infections. We defined a COVID-19 hospital admission as being hospitalised within 14 days following a positive RT-PCR test for SARS-CoV-2, including those who tested positive within 2 days of hospitalisation, or those who were hospitalised with an admission diagnosis of COVID-19 (appendix p 2). COVID-19 related deaths were all-cause deaths occurring within 28 days after a positive test for SARS-CoV-2 that were registered with National Records Scotland and included death certification, or deaths with COVID-19 on the death certificate as the cause of death. However, the number of COVID-19 related deaths was too small (n<5) in children with asthma to enable us to carry out any statistical analysis, so we did not include them in this report.

### Statistical analysis

A Cox proportional hazard model was used to derive the hazard ratio (HR) and 95% CIs for the association between asthma control and COVID-19 hospital admissions and deaths. This model, with calendar time as the timescale, eliminates the need to model the underlying temporal trends, which are estimated as the baseline hazard. A spline of age, sex, socioeconomic status, other risk groups of interest (ie, those identified by the QCOVID algorithm), and number of non-asthma related hospitalisations within the 2-year period before March 1, 2020, were included as adjustments. Socioeconomic status was determined using the Scottish Index of Multiple Deprivation (SIMD).^18^ The SIMD classification is based on deprivation quintiles: quintile 1 refers to the most deprived and quintile 5 refers to the most affluent. SIMD was assigned according to residential postcode. Adjustment for previous hospitalisation was used as a marker of severity or health-care seeking behaviour. Similarly, the Cox proportional hazard models were fitted to estimate the association between markers of uncontrolled asthma and the outcome of COVID-19 hospital admission. All analyses were carried out in children aged 5–17 years old and stratified by two age groups (5–11 years *vs* 12–17 years). Rates are shown per 100 000 people.

A sensitivity analysis was carried out using a 1-year look back before March 1, 2020, for the two markers of uncontrolled asthma. Another sensitivity analysis was also conducted including both prednisolone and dexamethasone prescriptions as the marker of asthma control. We also conducted a sensitivity analysis only analysing those who tested positive for SARS-CoV-2 and measured the markers of uncontrolled asthma at the date of test to see if the risk of COVID-19 hospitalisation was higher in those with poorly controlled asthma following testing positive. This was to account for children with previous hospitalisation for asthma or previous oral corticosteroids prescribing after March 1, 2020, but before their SARS-CoV-2 infection. We also undertook a post-hoc sensitivity analysis including regional health board as an adjusted variable in the model to investigate the potential impact of clustering.

Both the Cox proportional hazards model and estimation of cumulative incidence (appendix pp 16–17) used sampling weights, which were used to correct for the size of the registered general practice population being greater than the population in Scotland (some due to individuals who had recently moved). These weights were derived by matching the age and sex numbers in the general practice data to the Scottish population data. This adjustment ensured that the denominators in the tables matched the Scottish population.

Analyses were carried out in R (version 3.6.1).

### Role of the funding source

The funders of the study had no role in study design, data collection, data analysis, data interpretation, or the writing of the report.

## Results

752 867 children in the EAVE II linked dataset who were aged 5–17 years old on March 1, 2020, were included in the analysis. 63 463 children (8·4%) had clinician-diagnosed-and-recorded asthma. Among children with asthma, 4339 (6·8%) had confirmed SARS-CoV-2 infections, of whom 67 (1·5%) were admitted to hospital with COVID-19 (among them, fewer than five were due to nosocomial infections). Intensive care unit admissions and deaths were rare in this population (nine overall), so we are unable to evaluate these more severe outcomes. Among the 689 404 children without asthma, 40 231 (5·8%) had confirmed SARS-CoV-2 infections, of whom 382 (0·9%) were admitted to hospital with COVID-19. The baseline characteristics for children with asthma stratified by markers of asthma control are available in the appendix (pp 6–7).

The number of children being tested for SARS-CoV-2, testing positive, and being admitted to hospital with COVID-19 per 100 000 children, were greater among children with asthma than among children without asthma (table 1). Children with poorly controlled asthma had higher rates of being tested and higher rates of COVID-19 hospital admissions compared with those with well controlled asthma or without asthma. However, the difference in SARS-CoV-2 test positivity was inconsistent between children with poorly controlled asthma and well controlled asthma (table 1).

**Table 1:** Testing for SARS-CoV-2, positive tests, and COVID-19 hospital admissions in children with asthma aged 5–17 years, stratified by markers of asthma control

	**Overall number**	**Tested for SARS-CoV-2**	Tested positive for SARS-CoV-2*	**Number admitted to hospital with COVID-19**†	**Number being tested per 100 000 children**	**Number testing positive per 100 000 children**	**Number admitted to hospital with COVID-19 per 100 000 children**
**Asthma**							
No	689 404	258 604	40 231 (15·56%)	382 (0·95%)	37 511·2	5835·6	55·4
Yes	63 463	28 460	4339 (15·25%)	67 (1·54%)	44 845·0	6837·1	105·6
**Asthma control (previous hospital admission**‡**)**							
No asthma	689 224	258 506	40 222 (15·56%)	382 (0·95%)	37 506·8	5835·8	55·4
Asthma without previous hospital admission	62 002	27 611	4250 (15·39%)	58 (1·36%)	44 532·4	6854·6	93·5
Asthma with previous hospital admission	1641	947	98 (10·35%)	9 (9·18%)	57 708·7	5972·0	548·4
**Asthma control (oral corticosteroids prescription**§**)**							
No asthma	676 617	252 130	39 518 (15·67%)	366 (0·93%)	37 263·3	5840·5	54·1
Asthma with 0 courses of oral corticosteroids	52 007	22 432	3577 (15·95%)	47 (1·31%)	43 132·7	6877·9	90·4
Asthma with 1 course of oral corticosteroids	15 998	7952	965 (12·14%)	15 (1·55%)	49 706·2	6032·0	93·8
Asthma with 2 courses of oral corticosteroids	4335	2287	267 (11·67%)	10 (3·75%)	52 756·6	6159·2	230·7
Asthma with ≥3 courses of oral corticosteroids	3910	2263	243 (10·74%)	11 (4·53%)	57 877·2	6214·8	281·3
**Asthma control (previous hospital admission**‡**or oral corticosteroids prescription**§**)**							
No asthma	676 522	252 083	39 514 (15·67%)	366 (0·93%)	37 261·6	5840·8	54·1
Asthma with 0–1 course of oral corticosteroids and no previous hospital admission	67 221	29 980	4493 (14·99%)	59 (1·31%)	44 599·2	6683·9	87·8
Asthma with ≥2 courses of oral corticosteroids or previous hospital admission	9124	5001	563 (11·26%)	24 (4·26%)	54 811·5	6170·5	263·0

Data are n or n (%) unless otherwise stated.

* Denominators are the numbers tested for SARS-CoV-2.

† Denominators are the numbers tested positive for SARS-CoV-2.

‡ Hospital admission for asthma within two-year period prior to March 1, 2020.

§ Oral steroids prescriptions for prednisolone in two-year period prior to March 1, 2020.

Children with asthma were found to be at an increased risk of COVID-19 hospital admission (adjusted HR 1·49 [95% CI 1·14–1·94]) compared with those without asthma. Using previous hospital admission for asthma as the marker of uncontrolled asthma, children with asthma and no hospital admission for asthma were at greater risk of COVID-19 hospital admission than children without asthma; children with previous hospital admission for asthma were at increased risk of COVID-19 hospital admission compared with both other groups (table 2). Using oral corticosteroid prescriptions in the preceding 24 months as the marker of uncontrolled asthma, the highest risk was observed in those with two courses of corticosteroids (table 2). In the age-stratified analysis, similar results were found in those aged 12–17 years, whereas the risk decreased with one course and was highest in those with three or more courses in those aged 5–11 years (table 2). A similar pattern was observed in the age-stratified analysis directly comparing children with poorly controlled asthma with children with well controlled asthma (appendix p 8).

**Table 2:** COVID-19 hospital admissions in children with different markers of asthma control defined in the 2 years before March 1, 2020, compared with those with no asthma

	**Children aged 5–17 years with COVID-19 hospital admission**	**Adjusted HR (95% CI) for children aged 5–17 years**	**Adjusted HR (95% CI) for children aged 5–11 years**	**Adjusted HR (95% CI) for children aged 12–17 years**
Using previous hospital admission for asthma as marker of uncontrolled asthma*				
No asthma	382	1 (ref)	1 (ref)	1 (ref)
Asthma without previous hospital admission	58	1·36 (1·02–1·80)	2·05 (1·35–3·12)	1·06 (0·73–1·54)
Asthma with previous hospital admission	9	6·40 (3·27–12·53)	3·78 (1·20–11·93)	10·04 (4·39–22·97)
Using previous prescribed oral corticosteroids as marker of uncontrolled asthma*				
No asthma	366	1 (ref)	1 (ref)	1 (ref)
Asthma with 0 courses of oral corticosteroids	47	1·34 (0·98–1.82)	2·18 (1·36–3·50)	1·03 (0·68–1·55)
Asthma with 1 course of oral corticosteroids	15	1·52 (0·90–2·57)	1·30 (0·61–2·79)	1·79 (0·88–3·67)
Asthma with 2 courses of oral corticosteroids	10	3·53 (1·87–6·67)	3·21 (1·31–7·87)	3·96 (1·61–9·73)
Asthma with ≥3 courses of oral corticosteroids	11	3·38 (1·84–6·21)	4·81 (2·33–9·92)	1·92 (0·60–6·10)

HRs were derived using cox proportional hazard model adjusting for age, sex, socioeconomic status, nine other risk groups of interest, and number of non-asthma hospital admissions within the 2 years before March 1, 2020. HR=hazard ratio.

* 2-year look back on both markers of uncontrolled asthma was from March 1, 2020.

The sensitivity analysis including both prednisolone and dexamethasone prescriptions as the marker of uncontrolled asthma (HR 1·34 [95% CI 0·98–1·83] for no courses, 1·49 [0·89–2·52] for one course, 3·82 [2·08–7·00] for two courses, and 3·32 [1·81–6·11] for three or more courses, compared with those without asthma), the sensitivity analysis using 1-year look back before March 1, 2020, for both markers of asthma control (table 3), the subset analysis of those who tested positive for SARS-CoV-2 measuring the markers of uncontrolled asthma at the date of test (appendix p 9), and the post-hoc sensitivity analysis including regional health board as an adjusted variable in the model (appendix p 12), all showed similar results to the primary analysis. In our study, when focusing on COVID-19 hospital admission with more than 1 day length of hospital stay or focusing on COVID-19 hospital admission with previous positive test, the results did not differ much (appendix pp 10–11). The full models of HR for COVID-19 hospitalisation using both markers of uncontrolled asthma are available in appendix pp 13–14.

**Table 3:** COVID-19 hospital admissions in children with different markers of asthma control defined in the 1 year before March 1, 2020, compared with those with no asthma

	**Children aged 5–17 years with COVID-19 hospital admission**	**Adjusted HR (95% CI) for children aged 5–17 years**	**Adjusted HR (95% CI) for children aged 5–11 years**	**Adjusted HR (95% CI) for children aged 12–17 years**
Using previous hospital admission for asthma as marker of uncontrolled asthma*				
No asthma	382	1 (ref)	1 (ref)	1 (ref)
Asthma without previous hospital admission	63	1·46 (1·11–1·92)	2·08 (1·38–3·15)	1·18 (0·83–1·69)
Asthma with previous hospital admission	<5	4·71 (1·74–12·74)	4·39 (1·08–17·84)	5·10 (1·24–20·97)
Using previous prescribed oral corticosteroids as marker of uncontrolled asthma*				
No asthma	372	1 (ref)	1 (ref)	1 (ref)
Asthma with 0 course of oral corticosteroids	54	1·41 (1·06–1·89)	2·14 (1·38–3·34)	1·12 (0·76–1·64)
Asthma with 1 course of oral corticosteroids	13	1·87 (1·07–3·27)	1·08 (0·40–2·93)	2·80 (1·42–5·50)
Asthma with 2 courses of oral corticosteroids	5	3·08 (1·26–7·50)	3·33 (1·05–10·54)	2·75 (0·67–11·32)
Asthma with ≥3 courses of oral corticosteroids	5	3·53 (1·45–8·60)	8·11 (3·28–20·04)	..†

HRs were derived using cox proportional hazard model adjusting for age, sex, socioeconomic status, nine other risk groups of interest, and number of non-asthma hospital admissions within the 2 years before March 1, 2020. HR=hazard ratio.

* 1-year look back on both markers of uncontrolled asthma was from March 1, 2020.

† Not estimated because no events occurred in this group.

Similar results were observed when focusing on children aged 12–15 years as in the analysis of children aged 5–17 years. We found that the rates of being tested, testing positive for SARS-CoV-2, and COVID-19 hospitalisation were greater among children with asthma compared with those without asthma (table 4). Children with poorly controlled asthma had a higher rate of COVID-19 hospital admission than those with well controlled asthma or without asthma.

**Table 4:** Testing for SARS-CoV-2, positive tests, and COVID-19 hospital admissions in children with asthma aged 12–15 years, stratified by markers of asthma control

	**Overall number**	**Tested for SARS-CoV-2**	**Tested positive for SARS-CoV-2**	**Number admitted to hospital with COVID-19**	**Number being tested per 100 000 children**	**Number testing positive per 100 000 children**	**Number admitted to hospital with COVID-19 per 100 000 children**
**Asthma**							
No	204 272	73 795	12 418	113	36 125·9	6079·1	55·3
Yes	24 116	10 241	1566	19	42 465·6	6493·6	78·8
Asthma control (previous hospital admission*)							
No asthma	204 248	73 785	12 418	113	36 125·2	6079·9	55·3
Asthma without previous hospital admission	23 780	10 035	1541	14	42 199·3	6480·2	58·9
Asthma with previous hospital admission	359	216	25	5	60 167·1	6963·8	1392·8
**Asthma control (oral corticosteroids prescription**†**)**							
No asthma	202 301	72 860	12 301	108	36 015·6	6080·5	53·4
Asthma with 0 courses of oral corticosteroids	20 820	8580	1338	11	41 210·4	6426·5	52·8
Asthma with 1 course of oral corticosteroids	3492	1658	224	7	47 480·0	6414·7	200·5
Asthma with 2 courses of oral corticosteroids	919	463	66	<5	50 380·8	7181·7	326·4
Asthma with ≥3 courses of oral corticosteroids	857	475	55	<5	55 425·9	6417·7	350·1
**Asthma control (oral steroids prescription**†**)**							
No asthma	202 301	72 860	12 301	108	36 015·6	6080·5	53·4
Asthma with 0–1 course of oral corticosteroids	24 311	10 238	1562	18	42 112·6	6425·1	74·0
Asthma with ≥2 courses of oral corticosteroids	1775	938	121	6	52 845·1	6816·9	338·0
Asthma control (previous hospital admission*or oral corticosteroids prescription†**)**							
No asthma	202 287	72 856	12 301	108	36 016·2	6081·0	53·4
Asthma with 0–1 course of oral corticosteroids and no previous hospital admission	24 139	10 141	1549	15	42 010·9	6417·0	62·1
Asthma with ≥2 courses of oral corticosteroids or previous hospital admission	1962	1039	134	9	52 956·2	6829·8	458·7

* Hospital admission for asthma within 2-years before March 1, 2020.

† Oral steroids prescriptions for prednisolone in two-year period prior to March 1, 2020.

Overall, this definition of asthma control would translate into 9124 children aged 5–17 years with previous asthma hospital admission or two or more courses of oral corticosteroids in Scotland during the study period who should be considered a priority for vaccination, which can be scaled up to approximately 109 488 children in the UK, assuming the same prevalence of poorly controlled asthma in the other nations of the UK (table 1). Focusing on children aged 12–15 years, this would translate into 1962 children in Scotland and 23 544 children across the UK who should be considered a priority for vaccination (table 4).

## Discussion

We found that children aged 5–17 years with poorly controlled asthma are at markedly increased (3–6 times higher) risk of COVID-19 hospital admission compared with those without asthma. This would translate into 9124 school-aged children in Scotland and 109 488 children in the UK with poorly controlled asthma (previous asthma hospital admission or ≥2 courses of oral corticosteroids) who should be considered a priority for vaccination. Although the HR was elevated, the overall risk of admission to hospital with SARS-CoV-2 in children with asthma was low (1 in 380 children with poorly controlled asthma were admitted to hospital with COVID-19).

To our knowledge, this is the first national, population-level study assessing the risk of SARS-CoV-2 infections and COVID-19 hospital admissions among school-aged children with markers of uncontrolled asthma. Several studies have investigated the association between asthma and hospital admissions related to other respiratory viruses;^19^, ^20^, ^21^ however, none of these investigated markers of asthma control. Our study has several strengths. We developed a national linked dataset and have created a platform that allowed rapid access to and analysis of data from routinely collected electronic health records and national databases. This study is therefore less susceptible to recall or misclassification bias than are studies that rely on primary data collection. We recently did a validation exercise of QCOVID comorbidities for the Scottish population using EAVE II.^22^ The use of a large population aided study power, facilitating estimation of HRs in different risk groups.

Our study has several limitations. It is noteworthy that there were small absolute numbers of COVID-19 hospital admissions, intensive care unit admissions, and deaths in children with asthma. These small numbers precluded the opportunity for further investigations into the more severe outcomes. It is reflective of the fact that in most cases, COVID-19 affects children less severely than adults. There was an absence of more granular data on the reason for admission. In UK primary care settings, asthma diagnosis in childhood is based on clinical assessments^23^ with tests rarely undertaken.^24^ We relied on surrogate markers of poorly or suboptimally controlled asthma, although our results were consistent across multiple measures. Reasons for emergency room attendance were not reliably recorded in Scotland's national accident and emergency dataset. Therefore, we were unable to use emergency room attendance as a marker for uncontrolled asthma. We might have missed some children who were treated with oral corticosteroids in the emergency room and who were subsequently discharged without being admitted to hospital. Asthma severity was not assessed via Global Initiative for Asthma (GINA) treatment step.^23^ In addition, although indicators of uncontrolled asthma were included, it is possible that asthma control might have changed over the 2-year study period, potentially resulting from changes in behaviours or difficulties with access to care during the course of the COVID-19 pandemic. However, our sensitivity analysis using a 1-year look back period showed similar findings. Regarding the adjustment of other risk groups (post-hoc analyses results available in the appendix pp 13–14), we only included nine risk groups that were defined by the QCOVID prediction algorithm^16^ (which was based on an adult population) and that resulted in at least five hospital admissions during the study period, so we might have missed some important paediatric risk groups (eg, those with cystic fibrosis). Children aged 5–17 years with poorly controlled asthma had an increased rate of being tested compared with children with well controlled asthma or without asthma. This might be because they might be more likely to be admitted to hospital and therefore more likely to have routine SARS–CoV-2 testing and screening in hospital than those with well controlled asthma or without asthma. In our study, when focusing on COVID-19 hospital admission with more than 1 day length of hospital stay or focusing on COVID-19 hospital admission with previous positive test, the results did not differ much from the primary analysis (appendix pp 10–11). There might also have been different health-care seeking behaviours and a lower threshold for COVID-19 admission (influenced by physician and hospital factors) in children with poorly controlled asthma, which might have resulted in greater chances of being tested for SARS-CoV-2. This probably explains why our results suggested that children aged 5–11 years have a higher risk for COVID-19 hospital admission, even when they have not had previous hospital admission and when they had not had any courses of corticosteroids in the past 2 years. There could also be influences from mask policy and general instructions to the population on how to distance and behave in school during the study period. Due to mask policy becoming less stringent in the UK, the baseline risk increases and the benefits of vaccination might also increase. Our analysis was not able to include some potentially important confounders (such as tobacco exposure, unsuitable housing, or ethnicity) due to the lack of reliable recording of these social variables within electronic health records, with the consequence that residual confounding remains a possibility. Moreover, we did not have data on initial copy detection threshold and cycle number in our extracts of RT-PCR results. There was the possibility that these might have changed over the study period, thereby potentially affecting the case definition. As far as we are aware, there were no such changes in Scotland over the study period.

Similar findings have been reported elsewhere. GINA has reported that people with well controlled asthma do not appear to be at substantially increased risk of being infected with SARS-CoV-2 or of having severe COVID-19, but the risk of COVID-19 death was increased in people who recently needed oral corticosteroids for their asthma.^23^ The International Severe Acute Respiratory and emerging Infection Consortium WHO Clinical Characterisation Protocol UK study reported that 74 children (aged <16 years) with asthma were admitted to hospital with COVID-19. The authors noted that the proportion of children with asthma admitted to hospital with COVID-19 was higher than the national average. Of the 74 children studied, ten were transferred to critical care; none died.^25^ Our study has added robust and generalisable evidence using population level data and quantified associations between poorly controlled asthma and COVID-19 hospital admission. Building on this work, it is important for more detailed characterisation of markers of asthma control for severe COVID-19 outcomes in children and to investigate underlying mechanisms that predispose such children to these increased risks. This analysis also underscores the importance of maintaining good asthma control and careful monitoring of children with poorly controlled asthma if they develop SARS-CoV-2 infection. Good asthma control could help to protect children from developing more severe manifestations of COVID-19. With vaccines in children and young people being given and planned internationally and nationally, together with other public health surveillance data, policy makers will be able to use data from our study to inform decisions on vaccination priorities among school-aged children with asthma. This is particularly important considering the potential limited vaccine supplies and the lower absolute risk of serious COVID-19 in children.

In summary, we provide national evidence that in children and young people aged 5–17 years, markers of uncontrolled asthma were associated with an increased risk of COVID-19 hospital admission in Scotland. The findings from this linkage of multiple data sources have helped inform the prioritisation of school-aged children with poorly controlled asthma for vaccines.^26^

## Data sharing

All code used in this study is publicly available online. The data used in this study are sensitive due to individual patient-level data and will not be made publicly available. A data dictionary covering the datasets used in this study can be found at https://github.com/EAVE-II/EAVE-II-data-dictionary. All code developed for this analysis is available in our GitHub area: https://github.com/EAVE-II/Covid-asthma-children. We will also deposit the meta-data information in Health Data Research Innovation Gateway.

## Declaration of interests

AS and CR are members of the Scottish Government's Chief Medical Officer's COVID-19 Advisory Group. AS and CR are members of the New and Emerging Respiratory Virus Threats Advisory Group risk stratification subgroup. CR is a member of the Scientific Pandemic Influenza Group on Modelling. AS is a member of AstraZeneca's Thrombotic Thrombocytopenic Advisory Group and the Scottish Government's Standing Committee on Pandemics. SVK is co-chair of the Scottish Government's Expert Reference Group on Ethnicity and COVID-19. All roles are unremunerated. All other authors declare no competing interests.
