# Supplementary material for: Risk of COVID-19 hospital admission among children aged 5–17 years with asthma in Scotland: a national incident cohort study
Source: Lancet Respir Med. 2022 Feb;10(2):191–8. doi: 10.1016/S2213-2600(21)00491-4 (PMC8631918; doi:10.1016/S2213-2600(21)00491-4)
Supplement: Supplementary appendix [file mmc1.pdf]

THE LANCET
Respiratory Medicine

Supplementary appendix

This appendix formed part of the original submission and has been peer reviewed. We post it as supplied by the authors.

Shi T, Pan J, Katikireddi SV, et al. Risk of COVID-19 hospital admission among children aged 5–17 years with asthma in Scotland: a national incident cohort study. *Lancet Respir Med* 2021; published online Nov 30. https://doi.org/10.1016/S2213-2600(21)00491-4.

Supplementary material

Box 1: Predictor variables in the QCOVID algorithm

- accommodation (homeless, care home, neither)
- asthma
- atrial fibrillation
- blood cancer
- body mass index (BMI)
- cerebral palsy
- chronic kidney disease
- cirrhosis of liver
- congenital heart disease
- congestive cardiac failure
- chronic obstructive pulmonary disease (COPD)
- coronary heart disease
- dementia
- diabetes 1
- diabetes 2
- epilepsy
- ethnicity
- learning disability
- osteoporotic fracture
- Parkinson’s disease
- peripheral vascular disease
- pulmonary hypertension or pulmonary fibrosis
- rare neurological conditions
- rare pulmonary diseases
- respiratory cancer
- rheumatoid arthritis or systemic lupus erythematosus
- severe mental illness
- sickle cell disease
- stroke
- venous thromboembolism

Table S1: ICD-10 codes for COVID-19 illness

Code	Description
U07.1	COVID-19, virus identified
U07.2	COVID-19, virus not identified
Source: https://www.who.int/classifications/icd/COVID-19-coding-icd10.pdf	

ICD-10: International Classification of Diseases 10.

Table S2: List of 9 other risk groups of interest included in this study (using a cut-off of at least 5 events of COVID-19 hospitalisations)

Risk group of interest*	Coding
Blood cancer	Yes/no
Cerebral palsy	Yes/no
Congenital heart disease	Yes/no
Diabetes type 1	Yes/no
Epilepsy	Yes/no
Learning disability	Yes without Down's syndrome/yes with Down's syndrome/no
Osteoporotic fracture	Yes/no
Severe mental illness	Yes/no
Sickle cell disease	Yes/no

*Body mass index data were missing on 93.1% and ethnicity data were missing on 31.1% of participants; these variables were therefore not included.

Table S3: Reporting STROBE checklist for cohort studies

	Item No	Recommendation	Location
**Title and abstract**	1	(*a*) Indicate the study's design with a commonly used term in the title or the abstract	Page 2
		(*b*) Provide in the abstract an informative and balanced summary of what was done and what was found	Page 2
**Introduction**			
Background/rationale	2	Explain the scientific background and rationale for the investigation being reported	Page 4
Objectives	3	State specific objectives, including any prespecified hypotheses	Page 4
**Methods**			
Study design	4	Present key elements of study design early in the paper	Page 4
Setting	5	Describe the setting, locations, and relevant dates, including periods of recruitment, exposure, follow-up, and data collection	Page 4
Participants	6	(*a*) Give the eligibility criteria, and the sources and methods of selection of participants. Describe methods of follow-up	Page 4
		(*b*) For matched studies, give matching criteria and number of exposed and unexposed	NA
Variables	7	Clearly define all outcomes, exposures, predictors, potential confounders, and effect modifiers. Give diagnostic criteria, if applicable	Page 5
Data sources/ measurement	8*	For each variable of interest, give sources of data and details of methods of assessment (measurement). Describe comparability of assessment methods if there is more than one group	Page 5
Bias	9	Describe any efforts to address potential sources of bias	Page 5
Study size	10	Explain how the study size was arrived at	Page 5
Quantitative variables	11	Explain how quantitative variables were handled in the analyses. If applicable, describe which groupings were chosen and why	Page 5
Statistical methods	12	(*a*) Describe all statistical methods, including those used to control for confounding	Page 5-6
		(*b*) Describe any methods used to examine subgroups and interactions	Page 5-6
		(*c*) Explain how missing data were addressed	Page 5
		(*d*) If applicable, explain how loss to follow-up was addressed	NA
		(*e*) Describe any sensitivity analyses	NA
**Results**			
Participants	13*	(a) Report numbers of individuals at each stage of study—eg numbers potentially eligible, examined for eligibility, confirmed eligible, included in the study, completing follow-up, and analysed	Page 7
		(b) Give reasons for non-participation at each stage	NA
		(c) Consider use of a flow diagram	Page 5, Figure S1
Descriptive data	14*	(a) Give characteristics of study participants (eg demographic, clinical, social) and information on exposures and potential confounders	Page 7
		(b) Indicate number of participants with missing data for each variable of interest	Page 5, Table S2
		(c) Summarise follow-up time (eg, average and total amount)	Page 5

Outcome data	15*	Report numbers of outcome events or summary measures over time	Page 7
Main results	16	(*a*) Give unadjusted estimates and, if applicable, confounder-adjusted estimates and their precision (eg, 95% confidence interval). Make clear which confounders were adjusted for and why they were included	Page 7
		(*b*) Report category boundaries when continuous variables were categorized	NA
		(*c*) If relevant, consider translating estimates of relative risk into absolute risk for a meaningful time period	NA
Other analyses	17	Report other analyses done—eg analyses of subgroups and interactions, and sensitivity analyses	Page 7-8
**Discussion**			
Key results	18	Summarise key results with reference to study objectives	Page 8
Limitations	19	Discuss limitations of the study, taking into account sources of potential bias or imprecision. Discuss both direction and magnitude of any potential bias	Page 8-9
Interpretation	20	Give a cautious overall interpretation of results considering objectives, limitations, multiplicity of analyses, results from similar studies, and other relevant evidence	Page 9
Generalisability	21	Discuss the generalisability (external validity) of the study results	Page 9
**Other information**			
Funding	22	Give the source of funding and the role of the funders for the present study and, if applicable, for the original study on which the present article is based	Page 10

*Give information separately for exposed and unexposed groups. STROBE: Strengthening the Reporting of Observational studies in Epidemiology. NA: not applicable.

Table S4: Baseline characteristics of the included population

Variable	Level	Overall number	With asthma	Asthma with no prior hospitalisation*	Asthma with prior hospitalisation*	Asthma with 0 oral steroid^	Asthma with 1 oral steroid^	Asthma with 2 oral steroids^	Asthma with ≥3 oral steroids^
Total	Total	752867	63463 (8.4)	62002 (8.2)	1641 (0.2)	52007 (6.9)	15998 (2.1)	4335 (0.6)	3910 (0.5)
Age	5-11 years old	416713	25967 (6.2)	24973 (6)	1142 (0.3)	19219 (4.6)	10974 (2.6)	3033 (0.7)	2667 (0.6)
Age	12-17 years old	336154	37495 (11.2)	37029 (11)	499 (0.1)	32788 (9.8)	5024 (1.5)	1302 (0.4)	1243 (0.4)
Prior hospitalisation$	0	675385	54471 (8.1)	53581 (7.9)	1000 (0.1)	45660 (6.8)	12743 (1.9)	3216 (0.5)	2496 (0.4)
Prior hospitalisation	1+	77482	8992 (11.6)	8421 (10.9)	641 (0.8)	6347 (8.2)	3256 (4.2)	1119 (1.4)	1413 (1.8)
Sex	Female	367680	26364 (7.2)	25769 (7)	667 (0.2)	21779 (5.9)	6583 (1.8)	1667 (0.5)	1540 (0.4)
Sex	Male	385187	37099 (9.6)	36234 (9.4)	974 (0.3)	30228 (7.8)	9416 (2.4)	2668 (0.7)	2370 (0.6)
SIMD#	1 - High	167049	15908 (9.5)	15407 (9.2)	553 (0.3)	12636 (7.6)	4208 (2.5)	1181 (0.7)	1118 (0.7)
SIMD	2	146036	13265 (9.1)	12930 (8.9)	380 (0.3)	10772 (7.4)	3408 (2.3)	917 (0.6)	821 (0.6)
SIMD	3	135285	11595 (8.6)	11362 (8.4)	271 (0.2)	9536 (7)	2830 (2.1)	769 (0.6)	674 (0.5)
SIMD	4	147272	11566 (7.9)	11355 (7.7)	236 (0.2)	9683 (6.6)	2846 (1.9)	750 (0.5)	664 (0.5)
SIMD	5 - Low	150054	10617 (7.1)	10451 (7)	185 (0.1)	8946 (6)	2598 (1.7)	690 (0.5)	606 (0.4)
SIMD	NA	7172	512 (7.1)	497 (6.9)	17 (0.2)	434 (6.1)	109 (1.5)	28 (0.4)	27 (0.4)
Blood cancer	Yes	519	46 (8.9)	46 (8.9)	0 (0)	43 (8.3)	13 (2.5)	<5	<5
Cerebral palsy	Yes	1385	140 (10.1)	136 (9.8)	<5	117 (8.4)	31 (2.2)	13 (0.9)	14 (1)
Congenital heart disease	Yes	5874	633 (10.8)	618 (10.5)	19 (0.3)	516 (8.8)	164 (2.8)	40 (0.7)	55 (0.9)
Diabetes type 1	Yes	2481	260 (10.5)	254 (10.2)	6 (0.2)	229 (9.2)	39 (1.6)	7 (0.3)	15 (0.6)
Epilepsy	Yes	4465	492 (11)	476 (10.7)	17 (0.4)	398 (8.9)	117 (2.6)	30 (0.7)	49 (1.1)
Learning disability	Yes, excluding Downs syndrome	29616	3467 (11.7)	3380 (11.4)	91 (0.3)	2843 (9.6)	741 (2.5)	217 (0.7)	214 (0.7)
Learning disability	Yes - Downs syndrome	694	43 (6.2)	43 (6.2)	<5	39 (5.6)	24 (3.5)	<5	5 (0.7)
Osteoporotic fracture	Yes	17789	2203 (12.4)	2150 (12.1)	55 (0.3)	1834 (10.3)	406 (2.3)	114 (0.6)	93 (0.5)
Severe mental illness	Yes	1463	245 (16.7)	244 (16.7)	<5	212 (14.5)	32 (2.2)	7 (0.5)	11 (0.8)

Sickle cell disease	Yes	400	46 (11.5)	45 (11.2)	<5	38 (9.5)	10 (2.5)	5 (1.2)	10 (2.5)

Data are n (%); for %, overall number (3rd column) was used as denominator. * Hospitalisation for asthma within two-year period prior to March 1, 2020. ^ Oral steroids prescriptions for prednisolone in two-year period prior to March 1, 2020. $ Number of non-asthma hospitalisations within two-year period prior to March 1, 2020. # 1 indicates most deprived, 5 indicates least deprived. SIMD: Scottish Index of Multiple Deprivation

Table S5: Hazard ratio for COVID-19 hospitalisation comparing those with poorly controlled asthma and those with well controlled asthma (defined in the two years prior to March 1, 2020) in children

**Risk group**	**Number of covid hospitalisations 5-17 years**	**5-17 years adjusted HR (95% CI)**	**5-11 years adjusted HR (95% CI)**	**12-17 years adjusted HR (95% CI)**
Use prior hospitalisation for asthma as marker of uncontrolled asthma*				
Asthma without prior hospitalisation	58	1	1	1
Asthma with prior hospitalisation	9	4.24 (2.00-8.96)	1.75 (0.51-6.00)	10.41 (4.17-25.99)
Use previous prescribed oral steroids as marker of uncontrolled asthma*				
Asthma with 0 course of oral steroids	47	1	1	1
Asthma with 1 course of oral steroids	15	0.97 (0.53-1.8)	0.53 (0.22-1.28)	1.78 (0.79-3.98)
Asthma with 2 courses of oral steroids	10	2.43 (1.21-4.86)	1.51 (0.59-3.90)	4.08 (1.53-10.9)
Asthma with ≥3 courses of oral steroids	11	2.09 (1.04-4.20)	1.81 (0.76-4.29)	1.80 (0.52-6.22)

HR: Hazard Ratio. CI: Confidence Interval. Hazard ratios were derived using cox proportional hazard model adjusting for age, sex, socioeconomic status, nine other risk groups of interest, and number of non-asthma hospitalisations within two-year period prior to March 1, 2020. * Two-year look back on both markers of uncontrolled asthma was from March 1, 2020.

Table S6: Subset analysis of hazard ratio for COVID-19 hospitalisation among 5-17 years old who were tested positive

Risk group	Number of covid hospitalisations	Adjusted HR (95% CI)
Use prior hospitalisation for asthma as marker of uncontrolled asthma*		
No asthma	361	1
Asthma without prior hospitalisation	59	1.46 (1.1-1.93)
Asthma with prior hospitalisation	7	7.08 (3.28-15.3)
Use previous prescribed oral steroids as marker of uncontrolled asthma*		
No asthma	338	1
Asthma with 0 course of oral steroids	40	1.3 (0.93-1.82)
Asthma with 1 course of oral steroids	17	1.52 (0.93-2.5)
Asthma with 2 courses of oral steroids	14	4.62 (2.68-7.97)
Asthma with ≥3 courses of oral steroids	18	3.83 (2.36-6.23)

The Hazard ratio compares those with different markers of asthma control (defined in the two years prior to March 1, 2020) and those with no asthma in children. Hazard ratios were derived using cox proportional hazard model adjusting for age, sex, socioeconomic status, nine other risk groups of interest, number of non-asthma hospitalisations within two-year period prior to March 1, 2020, and the time from March 1, 2020 to the date of infection (this is to adjust for different waves/dominant variants at baseline). * Two-year look back on both markers of uncontrolled asthma was from the date of infection.

Table S7: Sensitivity analysis of hazard ratio for COVID-19 hospitalisation among 5-17 years old, focusing on >1 day length of hospital stay

**Risk group**	**Number of covid hospitalisations**	**Adjusted HR (95% CI)**
Use prior hospitalisation for asthma as marker of uncontrolled asthma*		
No asthma	153	1
Asthma without prior hospitalisation	23	1.27 (0.82,1.98)
Asthma with prior hospitalisation	3	5.11 (1.61,16.23)
Use previous prescribed oral steroids as marker of uncontrolled asthma*		
No asthma	144	1
Asthma with 0 course of oral steroids	19	1.3 (0.8,2.11)
Asthma with 1 course of oral steroids	7	1.79 (0.83,3.84)
Asthma with 2 courses of oral steroids	4	3.5 (1.28,9.53)
Asthma with ≥3 courses of oral steroids	5	3.57 (1.42,8.97)

The Hazard ratio compares those with different markers of asthma control (defined in the two years prior to March 1, 2020) and those with no asthma in children. Hazard ratios were derived using cox proportional hazard model adjusting for age, sex, socioeconomic status, nine other risk groups of interest, and number of non-asthma hospitalisations within two-year period prior to March 1, 2020. * Two-year look back on both markers of uncontrolled asthma was from March 1, 2020.

Table S8: Sensitivity analysis of hazard ratio for COVID-19 hospitalisation among 5-17 years old, focusing on those with prior positive test

**Risk group**	**Number of covid hospitalisations**	**Adjusted HR (95% CI)**
Use prior hospitalisation for asthma as marker of uncontrolled asthma*		
No asthma	168	1
Asthma without prior hospitalisation	28	1.45 (0.97,2.17)
Asthma with prior hospitalisation	4	7.78 (2.86,21.18)
Use previous prescribed oral steroids as marker of uncontrolled asthma*		
No asthma	165	1
Asthma with 0 course of oral steroids	22	1.33 (0.85,2.08)
Asthma with 1 course of oral steroids	5	1.24 (0.51,3.01)
Asthma with 2 courses of oral steroids	3	2.69 (0.85,8.52)
Asthma with ≥3 courses of oral steroids	5	4.09 (1.64,10.25)

The Hazard ratio compares those with different markers of asthma control (defined in the two years prior to March 1, 2020) and those with no asthma in children. Hazard ratios were derived using cox proportional hazard model adjusting for age, sex, socioeconomic status, nine other risk groups of interest, and number of non-asthma hospitalisations within two-year period prior to March 1, 2020. * Two-year look back on both markers of uncontrolled asthma was from March 1, 2020.

Table S9: Sensitivity analysis of hazard ratio for COVID-19 hospitalisation among 5-17 years old, adjusting for Health Board

**Risk group**	**Adjusted HR (95% CI)**
Use prior hospitalisation for asthma as marker of uncontrolled asthma*	
No asthma	1
Asthma without prior hospitalisation	1.36 (1.03-1.81)
Asthma with prior hospitalisation	6.46 (3.3-12.65)
Use previous prescribed oral steroids as marker of uncontrolled asthma*	
No asthma	1
Asthma with 0 course of oral steroids	1.35 (0.99-1.84)
Asthma with 1 course of oral steroids	1.52 (0.9-2.56)
Asthma with 2 courses of oral steroids	3.45 (1.83-6.52)
Asthma with ≥3 courses of oral steroids	3.27 (1.78-6.02)

This was a post-hoc analysis. The Hazard ratio compares those with different markers of asthma control (defined in the two years prior to March 1, 2020) and those with no asthma in children. Hazard ratios were derived using cox proportional hazard model adjusting for age, sex, socioeconomic status, nine other risk groups of interest, Health Board and number of non-asthma hospitalisations within two-year period prior to March 1, 2020. * Two-year look back on both markers of uncontrolled asthma was from March 1, 2020.

Table S10: Full model of hazard ratio for COVID-19 hospitalisation among 5-17 years old, using previous prescribed oral steroids as marker of uncontrolled asthma

Variable	Level	Adjusted HR (95% CI)
Age	Linear	1.1 (1.07-1.13)
	Nonlinear	NA
Sex	Female	1
	Male	0.74 (0.61-0.89)
SIMD	1 - High	1
	2	0.75 (0.57-0.97)
	3	0.69 (0.52-0.91)
	4	0.69 (0.53-0.91)
	5 - Low	0.45 (0.32-0.62)
	NA	0.68 (0.25-1.85)
Prior hospitalisation$	0	1
	1+	3.64 (2.95-4.48)
Blood cancer	Yes	9.95 (4.38-22.6)
Cerebral palsy	Yes	2.44 (1.03-5.74)
Congenital heart disease	Yes	1.4 (0.69-2.87)
Diabetes type 1	Yes	2.51 (1.18-5.37)
Epilepsy	Yes	3.06 (1.8-5.2)
Learning disability	Yes, excluding Downs syndrome	1.32 (0.91-1.92)
Learning disability	Yes - Downs syndrome	3.37 (0.99-11.5)
Osteoporotic fracture	Yes	1.54 (0.98-2.42)
Severe mental illness	Yes	2.26 (0.92-5.59)
Sickle cell disease	Yes	10.02 (4.09-24.57)
Previous prescribed oral steroids*	No asthma	1
	Asthma with 0 course of oral steroids	1.34 (0.98-1.82)
	Asthma with 1 course of oral steroids	1.52 (0.9-2.57)
	Asthma with 2 courses of oral steroids	3.53 (1.87-6.67)
	Asthma with ≥3 courses of oral steroids	3.38 (1.84-6.21)

This was a post-hoc analysis. The Hazard ratio compares those with different markers of asthma control (defined in the two years prior to March 1, 2020) and those with no asthma in children. Hazard ratios were derived using cox proportional hazard model adjusting for age, sex, socioeconomic status, nine other risk groups of interest and number of non-asthma hospitalisations within two-year period prior to March 1, 2020. * Two-year look back on both markers of uncontrolled asthma was from March 1, 2020. $ Number of non-asthma hospitalisations within two-year period prior to March 1, 2020. # 1 indicates most deprived, 5 indicates least deprived. SIMD: Scottish Index of Multiple Deprivation.

Table S11: Full model of hazard ratio for COVID-19 hospitalisation among 5-17 years old, using prior hospitalisation for asthma as marker of uncontrolled asthma

Variable	Level	Adjusted HR (95% CI)
Age	Linear	1.1 (1.07-1.13)
	Nonlinear	NA
Sex	Female	1
	Male	0.74 (0.61-0.9)
SIMD	1 - High	1
	2	0.75 (0.57-0.97)
	3	0.69 (0.52-0.92)
	4	0.7 (0.53-0.92)
	5 - Low	0.45 (0.32-0.62)
	NA	0.67 (0.25-1.84)
Prior hospitalisation$	0	1
	1+	3.72 (3.02-4.57)
Blood cancer	Yes	9.88 (4.35-22.46)
Cerebral palsy	Yes	2.42 (1.03-5.71)
Congenital heart disease	Yes	1.41 (0.69-2.88)
Diabetes type 1	Yes	2.49 (1.17-5.31)
Epilepsy	Yes	3.07 (1.81-5.21)
Learning disability	Yes, excluding Downs syndrome	1.32 (0.91-1.92)
Learning disability	Yes - Downs syndrome	3.35 (0.98-11.4)
Osteoporotic fracture	Yes	1.54 (0.98-2.42)
Severe mental illness	Yes	2.27 (0.92-5.6)
Sickle cell disease	Yes	10.49 (4.28-25.72)
Prior hospitalisation for asthma	No asthma	1
	Asthma without prior hospitalisation	1.36 (1.02-1.8)
	Asthma with prior hospitalisation	6.4 (3.27-12.53)

This was a post-hoc analysis. The Hazard ratio compares those with different markers of asthma control (defined in the two years prior to March 1, 2020) and those with no asthma in children. Hazard ratios were derived using cox proportional hazard model adjusting for age, sex, socioeconomic status, nine other risk groups of interest and number of non-asthma hospitalisations within two-year period prior to March 1, 2020. * Two-year look back on both markers of uncontrolled asthma was from March 1, 2020. $ Number of non-asthma hospitalisations within two-year period prior to March 1, 2020. # 1 indicates most deprived, 5 indicates least deprived. SIMD: Scottish Index of Multiple Deprivation.

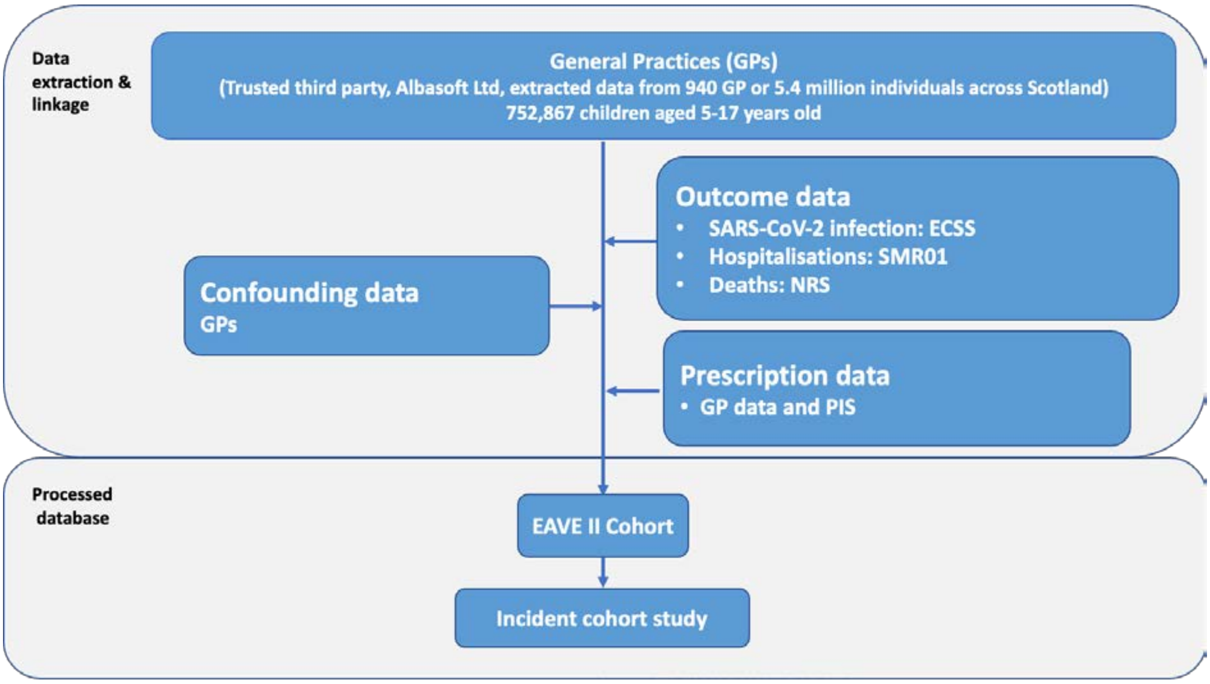

Community Health Index (CHI) numbers were used to link all datasets. ECSS: Electronic Communication of Surveillance in Scotland. SMR: Scottish Morbidity Record. NRS: National Records of Scotland. PIS: Prescribing Information System.

Figure S1: Data linkage diagram

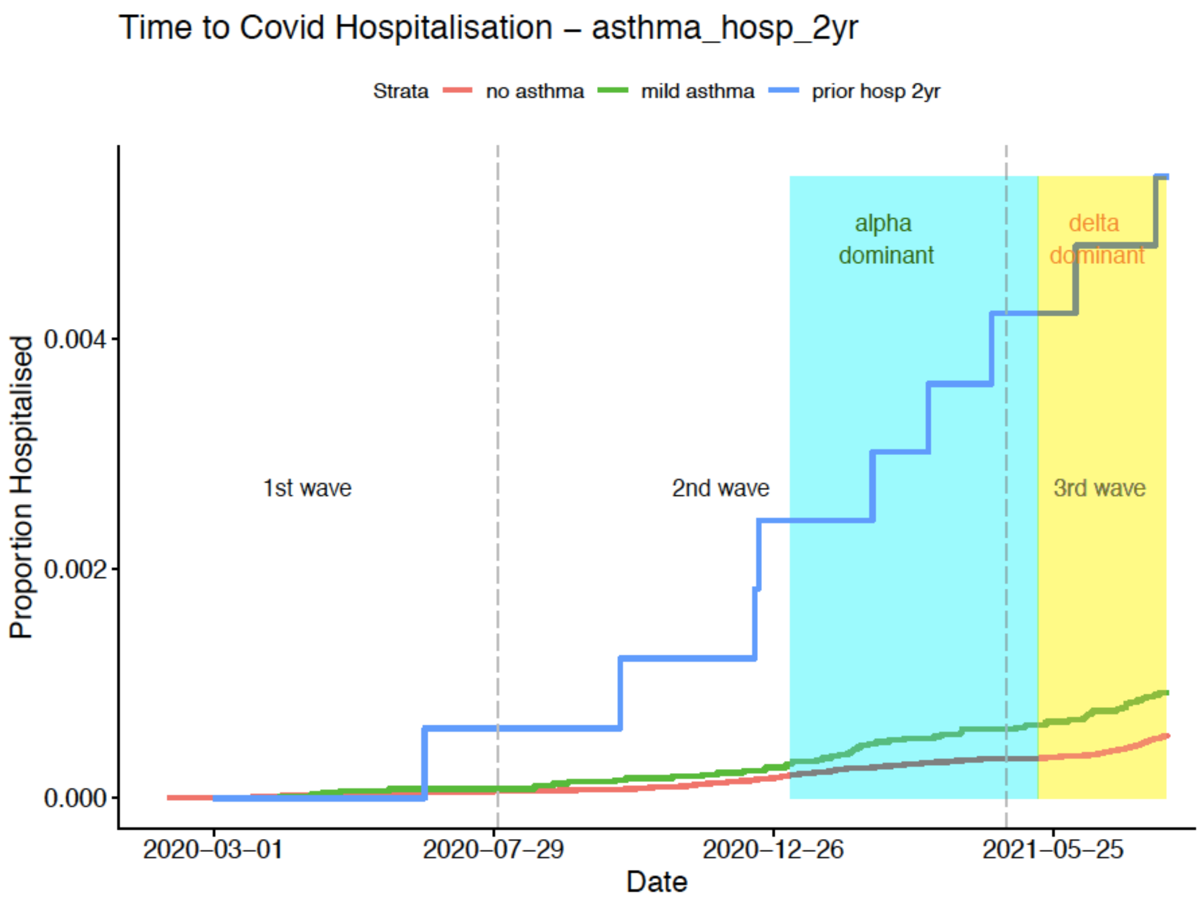
Time to Covid Hospitalisation – asthma_hosp_2yr
Strata
no asthma
mild asthma
prior hosp 2yr
Proportion Hospitalised
0.004
0.002
0.000
alpha dominant
delta dominant
1st wave
2nd wave
3rd wave
2020–03–01
2020–07–29
2020–12–26
2021–05–25
Date

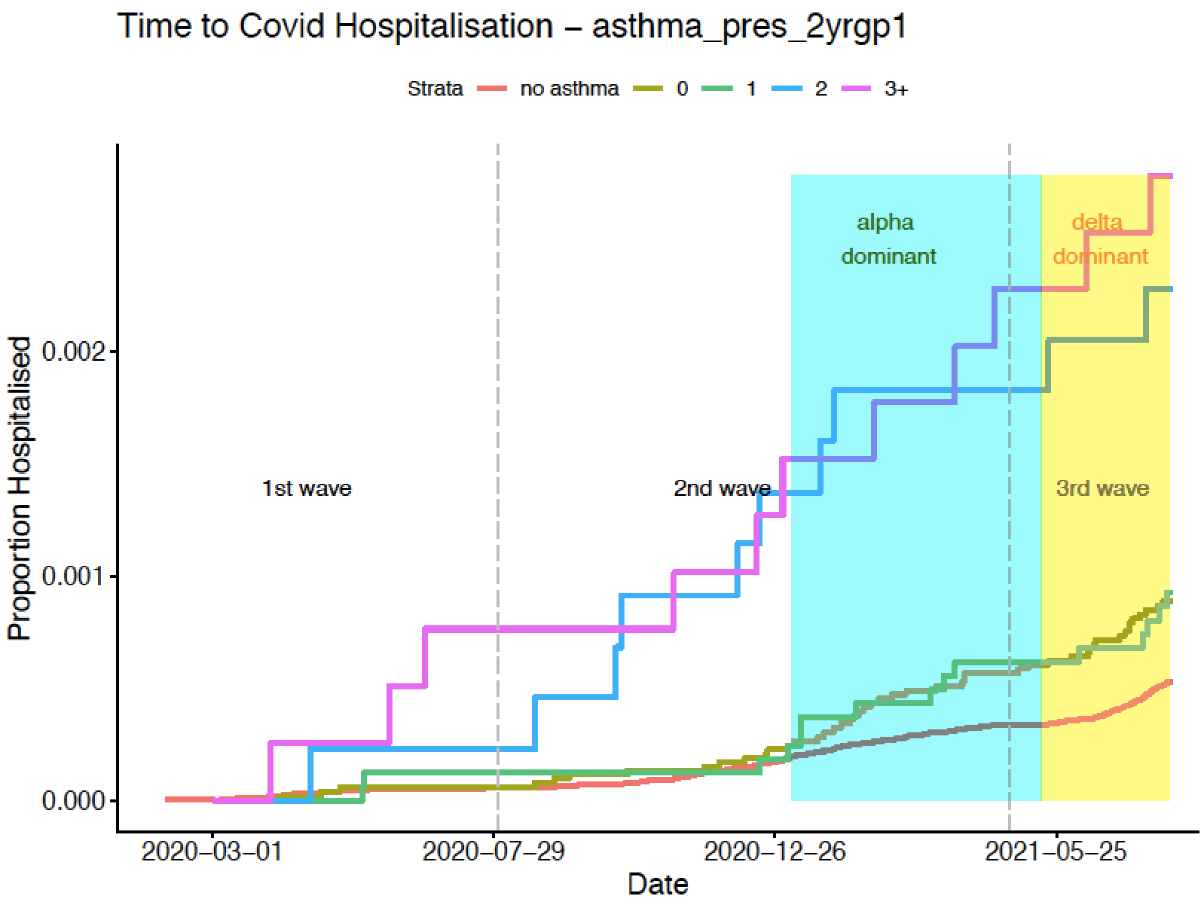
Time to Covid Hospitalisation – asthma_pres_2yrgp1
Strata
no asthma
0
1
2
3+
Proportion Hospitalised
0.002
0.001
0.000
alpha dominant
delta dominant
1st wave
2nd wave
3rd wave
2020–03–01
2020–07–29
2020–12–26
2021–05–25
Date

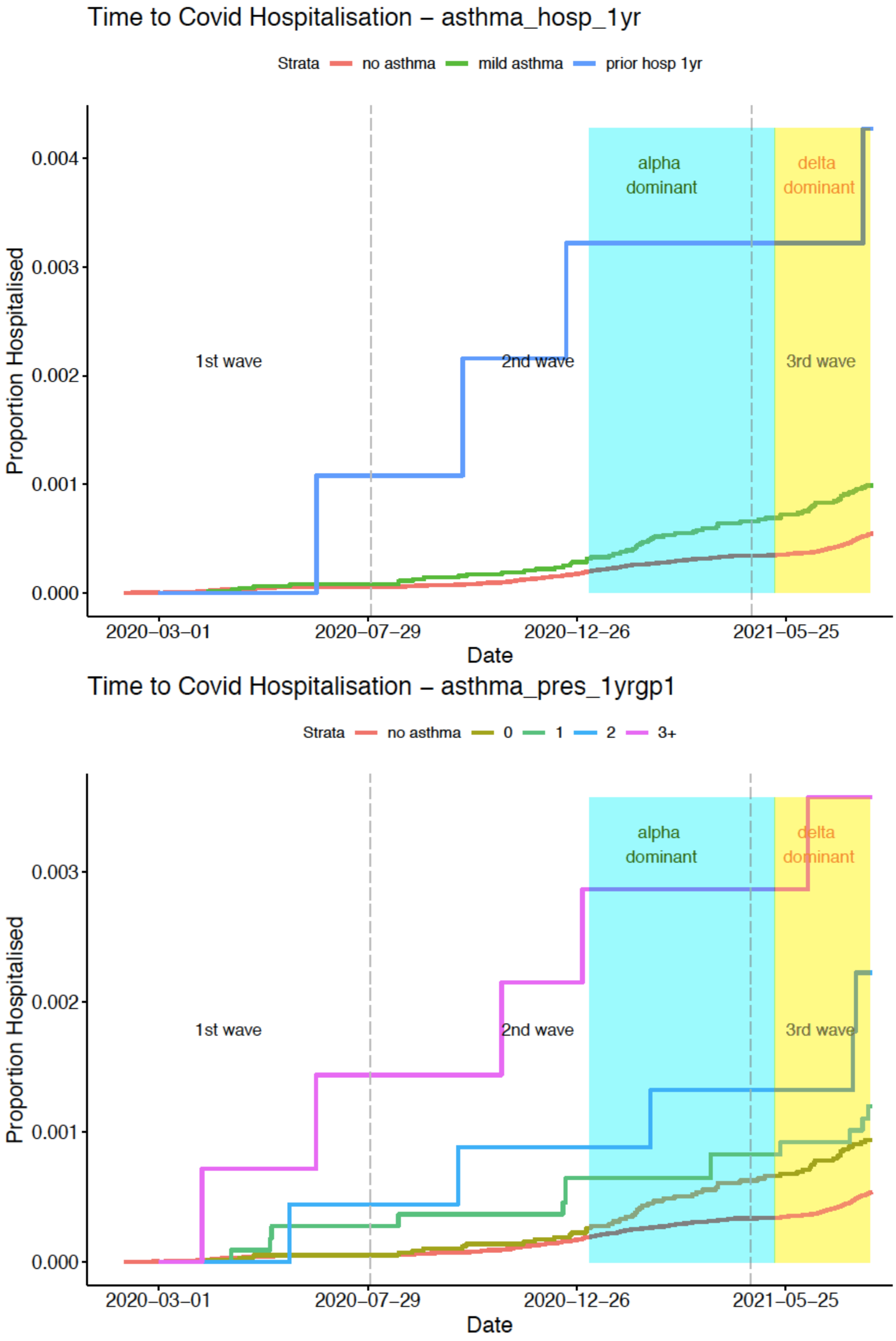

Figure S2: Time to COVID-19 hospitalisation over time among children with asthma aged 5 to 17 years old

The first two plots used prior hospitalisation for asthma or oral steroids prescriptions for prednisolone within two-year period prior to March 1, 2020 as the markers of uncontrolled asthma. The last two plots used prior hospitalisation for asthma or oral steroids prescriptions for prednisolone within one-year period prior to March 1, 2020 as the markers of uncontrolled asthma. The cumulative incidence of the outcome (COVID-19 hospitalisation) was plotted against the time and stratified by the presence or absence of asthma and markers of asthma control. The cumulative incidence was estimated using the Kaplan-Meier method. The time period in the plots was marked by different waves (first wave from March to July 2020, second wave from August 2020 to April 2021 and third wave from May to July 2021) and also by different dominant variants. Alpha variant of concern (VOC) became dominant by January 4, 2021 and Delta VOC became dominant by May 17, 2021. These curves generally show an increasing difference between children with poorly controlled asthma and well controlled asthma from the second wave onwards.
